# Selection of Cell Populations with High or Low Surface Marker Expression Using Magnetic Sorting

**DOI:** 10.3390/cells12091286

**Published:** 2023-04-29

**Authors:** Natalia Polyakova, Oleg Kandarakov, Alexander Belyavsky

**Affiliations:** 1 Engelhardt Institute of Molecular Biology, Russian Academy of Sciences, Moscow 119991, Russia; natae05@yandex.ru (N.P.); oleg.kandarakov@gmail.com (O.K.); 2 Institute of Higher Nervous Activity and Neurophysiology, Russian Academy of Sciences, Butlerova 5A, Moscow 117485, Russia

**Keywords:** MACS, magnetic selection, LNGFR, EGFP, dsRed-Express2, retroviral vectors, transduction, bicistronic constructs, cytometry

## Abstract

Magnetic cell sorting technology stands out because of its speed, simplicity, and ability to process large cell numbers. However, it also suffers from a number of drawbacks, in particular low discrimination power, which results in all-or-none selection outcomes limited to a bulk separation of cell populations into positive and negative fractions, as well as the modest purity of the selected cells and the inability to select subpopulations of cells with high expression of a surface marker. In the present study, we developed a simple solution to this problem and confirmed the effectiveness of this approach by multiple experiments with the magnetic selection of transduced cell populations. Murine NIH 3T3 cells were transduced with the bicistronic retroviral vector constructs co-expressing fluorescent reporter proteins EGFP (enhanced green fluorescent protein) or DsRed-Express 2 and LNGFR (low-affinity nerve growth factor receptor) as surface selection markers. The effects of the magnetic selection of transduced cells with anti-LNGFR Micro Bead (MB) doses ranging from 0.5 to 80 µL have been assessed. Low doses of MBs favored the depletion of weakly positive cells from the population, resulting in the higher expression levels of EGFP or DsRed-Express2 reporters in the selected cell fractions. Low MB doses also contributed to the increased purity of the selected population, even for samples with a low initial percentage of positive cells. At the same time, high MB doses resulted in the increased yield and a more faithful representation of the original expression profiles following selection. We further demonstrate that for populations with fairly narrow distribution of expression levels, it is possible to achieve separation into high- and low-expressing subsets using the two-stage selection scheme based on the sequential use of low and high doses of MBs. For populations with broad expression distribution, a one-stage selection with low or high doses of MBs is sufficient for a clear separation of low- and high-expressing subsets in the column-retained and flow-through fractions, respectively. This study substantially extends the potential of magnetic cell sorting, and may open new possibilities in a number of biomedical applications.

## 1. Introduction

The technology of magnetic cell selection (MACS, magnetic-activated cell sorting), proposed in the 1990s by Miltenyi et al. [1], eventually became one of the mainstays of mass cell sorting. Magnetic cell selection is currently employed in various research fields, as well as in medicine [2,3]. The advantages of the method include speed, simplicity, and the ability to select a large number of cells with high viability in a short time, all this without involvement of expensive equipment. However, as with any other method, it has its own disadvantages, which include a limited purity of selected cells [4,5,6,7], difficulties in selecting rare populations [8], or complicated cell sorting using two or more markers. One of the most significant disadvantages of the magnetic selection process has long been considered its low discrimination power, which resulted in all-or none selection outcomes, namely the bulk separation of cells into largely positive and negative fractions. The possibility of a more discriminating magnetic selection process has not been previously discussed in the literature.

It should be emphasized that these drawbacks of magnetic selection may in part be related to the conditions of cell selection [5,9,10,11,12]. For example, the study by Semple et al. [9] demonstrated that the decrease in the antibody amount during selection may reduce the yield of the selected cells, although the purity of cell selection remains relatively constant. Wang et al. [5] in a comparative study of four NK cell isolation protocols using both negative and CD49b-positive isolation kits from Miltenyi Biotec (Bergisch Gladbach, Germany) and Stemcell Technologies (Vancouver, BC, Canada) demonstrated a significant variation in the purity and viability of the isolated NK cells depending on the sets and markers used. The magnetic separation of the cell populations using alkaline phosphatase as selection marker showed that with an increase in the concentration of anti-ALPL-APC and/or anti-APC with magnetic microparticles, the yield of the selected cells increases [13]. At the same time, a comparison of positive and negative magnetic selection of monocytes showed that a two-fold decrease in the concentration of antibodies does not affect the purity, selectivity, and expression of CD14 in isolated monocytes [14].

The efficiency of the magnetic cell selection and the purity of the selected samples, defined as the ability to separate target cells from other cells and the percentage of undesired cells in selected samples, respectively, may depend on a number of factors. Currently, several solutions have been proposed to improve the efficiency of cell selection. As a solution to the problem of the magnetic selection of cells using two or more markers, Miltenyi Biotec developed the MACS MultiSort, as well as the REAlease technologies; two methods of multiparameter magnetic selection of cells. Alternative systems of magnetically activated cell sorting using microfluidics have been also developed [15,16,17]. A three-stage selection system has been proposed for the selection of rare populations by magnetic selection [8], while the combination of MACS and fluorescent cell sorting allows the selection and purification of complex and rare cellular subpopulations and, for example, is quite frequently used in hematology research [18]. However, approaches to magnetic selection of cell populations differing in marker expression levels, in particular highly expressing populations, have not yet been developed. Solving this problem may open the way to a simple and inexpensive mass selection of cells with high marker expression or even to a separation of high- and low-expressing cell subsets.

In the present study, we attempted to address this issue. We reasoned that magnetic microbeads (MBs) that attach to a selectable marker molecules expressed on cell surface are likely to be distributed according to the representation of this marker on cells. Therefore, under conditions of limited MB availability, cells with lower marker expression will be less heavily labeled by MBs, and thus, are more likely to pass through the magnet compared to the cells with higher expression. This in turn may result in the preferential isolation of high-expressing cell subsets. The experiments reported in this study provide thorough justification of this assumption.

## 2. Materials and Methods

### 2.1. Cell Cultures

NIH 3T3 and HEK 293 cells were cultured in DMEM (Dulbecco’s Modified Eagle’s Medium) (HyClone Laboratories, South Logan, UT, USA) with 4.5 g/L glucose, 10% fetal bovine serum (FBS) (HyClone Laboratories), 2 mM glutamine (Gibco, Gaithersburg, MD, USA), 100 units/mL penicillin, and 100 μg/mL streptomycin (Gibco) in 5% CO_2_ at 37 °C. Cells were detached with TrypLE Express (Gibco) treatment for 2–5 min at 37 °C. For magnetic selection and cytometry, the collected cells were centrifuged at 150× *g* for 10 min, the supernatant was removed, and the cells were suspended in phosphate buffer with 5 mM EDTA and 0.5% FBS (PBE), unless otherwise indicated.

### 2.2. Creation of Retroviral Expression Constructs

Bicistronic retroviral constructs with EGFP or DsRed-Express2 reporter protein ORFs in the 1st position and hLNGFR (human low-affinity nerve growth factor receptor) truncated ORF in the 2nd position were prepared using vector MigR1ad1. This vector was created on the basis of the original bicistronic MigR1 vector (Addgene #27490) [19] to incorporate an extended polylinker, and was prepared by insertion into Bgl II and Xho I sites of the MigR1 vector of the DNA fragment obtained by annealing oligos

Ad#MigR-S1 (GATCTGCGATCGCGGCCGCACGCGTATTAGGATCCAATTGCCTGCAGGC) and Ad#MigR-A1 (TCGAGCCTGCAGGCAATTGGATCCTAATACGCGTGCGGCCGCGATCGCA)

The hLNGFR (human low-affinity nerve growth factor receptor) truncated ORF (aa 1-274) lacking the intracellular domain was amplified from the plasmid p75-RFP (Addgene #24092) using KAPA HiFi DNA polymerase (Roche Sequencing and Life Science, Wilmington, MA, USA). The 1st PCR round was performed with oligonucleotides hLNGFR-S1m (GGTGCCACCGGCCGCGCTATGGAC) and hLNGFR-A1 (TCATTGTCGACCTATCACCTCTTGAAGGCTATGTAGGC) to eliminate the undesired Nco I site near the Start codon, while the 2nd PCR round was performed with primers hLNGFR-S2 (TCATTGTCGACCTATCACCTCTTGAAGGCTATGTAGGC) and hLNGFR-A1, followed by insertion of the final fragment into the 2nd position of the MigR1ad1 vector via Nco I and Sal I sites to replace the original EGFP sequence. The correctness of the inserted fragment was verified by sequencing. The resulting MigLNR2 vector was used to prepare the final expression constructs.

To create the pMigLNR2-EGFP construct, the EGFP ORF was re-cloned from the pLeGo-G2 plasmid [20] into the 1st position of the MigLNR2 vector via BamH I and EcoR I sites. To create the pMigLNR2-DsREx construct, the chemically synthesized (GenScript, Piscataway, NJ, USA) DsRed-Express2 [21] ORF was inserted into the 1st position of the MigLNR2 vector via the BamH I andEcoR I sites.

### 2.3. NIH 3T3 Cell Transduction

HEK 293 cells in a 10 cm Petri dish at 30–35% confluence were transfected by the calcium phosphate method. For the production of viral particles, a calcium phosphate solution with 10 µg of pMigLNR2-EGFP or pMigLNR2-DsREx expression plasmid and 10 µg pCL-Eco (Addgene#12371) packaging plasmid was added to the culture medium. After 24 h, unless otherwise indicated, the medium with virus-containing supernatants (VCS) was collected and centrifuged for 5 min at 150× *g*; then, the VCS was passed through a 0.45 µm filter (Corning, Glendale, AR, USA) and used for the transduction of NIH 3T3 cells.

NIH 3T3 cells were seeded in the wells of a 6-well plate with an initial density of 20,000 cells/cm^2^, and when 35–40% confluence was reached, the cells were transduced with various doses of VCS with the addition of 16 μg/mL of polybrene (Sigma-Aldrich Sigma-Aldrich St. Louis, MO, USA). The efficiency of the cell transduction was determined by cytometry analysis 2–3 days later based on the percentage of cells expressing the reporter protein in the population. The transduced cell cultures were cultured under the conditions described above.

### 2.4. Magnetic Selection of Transduced Cells

For the magnetic selection of the transduced cells, MACSelect anti-LNGFR Micro Beads (No. 130-091-330) from Miltenyi Biotec, hereinafter referred to as MBs, were used. After the incubation of cells with the above MBs, selection was carried out on LS columns (No. 130-042-401) or MS columns (No. 130-042-201), mounted on a QuadroMACS or OctoMACS Separators (Miltenyi Biotec), respectively, in accordance with the recommendations of the manufacturer. In brLSef, the transduced cells were detached with TrypLE Express (Gibco) solution for 5 min. The collected cells were centrifuged at 150× *g* for 10 min, the supernatant was removed, and the cells were suspended in 320 µL of PBE. Furthermore, different amounts of MBs were added to the suspension and incubated for 15 min on ice; the cell suspension was then brought to 2 mL and applied to a column mounted on a MACS Separator. The column was then washed with 12 mL or 2 mL of PBE buffer for LS or MS columns, respectively. The cells of the flow-through fraction were collected in a 15 mL tube. To collect the cells of the fraction retained on the column, the column was removed from the magnet and placed in a 15 mL tube. For LS or MS columns, 5 mL and 1 mL of PBE buffer were added, respectively, and the buffer was pushed through the column with a plunger, collecting cells into test tubes. The cells of the column-retained and flow-through fractions were counted using a hemocytometer and analyzed on a cytometer. A single-stage magnetic selection was performed with the LS columns. For the two-stage magnetic selection, the first stage was carried out with the LS column; the second one with the MS column. During the two-stage selection, the collected cells of the flow-through fraction were additionally incubated with MBs and selected on the MS column, as described above. If necessary, the selected cells were further cultured in the above medium. Cell viability was determined using Trypan blue. The cells were photographed with a Leica DMI 4000B microscope camera.

### 2.5. Flow Cytometry

The cells before and after the magnetic selection were collected as described above and stored for no more than 30 min at 4 °C before cytometry. The collected cells were analyzed on a BDLSR Fortessa cytometer (BD Life Sciences, Franklin Lakes, NJ, USA) equipped with a 488 nm laser with an emission filter of 530 ± 15 nm, a 561 nm laser with an emission filter of 586 ± 7.5 nm, and a 640 nm laser with an emission filter of 640 ± 7 nm, which were used to detect the fluorescence of EGFP, DsRed-Express2, and antibodies. To stain the cell samples with anti-LNGFR antibodies, the cells were washed twice in phosphate-buffered saline (PBS), resuspended in 100 µL of PBS containing 0.25 mM EDTA and 0.5% FBS, and incubated in the dark on ice for 40 min with the following antibodies (BioLegend, San Diego, CA, USA): Alexa Fluor 647-conjugated anti-human CD271 antibodies (clone ME20.4; catalogue no. 345114; 1:20) or FITC-conjugated anti-human CD271 (clone ME20.4; catalogue no. 345104; 1:20). Cell clusters and debris were eliminated by sequential gating of cells on size and granularity (FSC-A/SSC-A), and on area and peak height (FSC-A/FSC-H). Cytometric settings were kept unchanged during the measurement of all samples. The amount of data collected for each sample was at least 10,000 events. Cytometric data were processed using the Flowing Software program version 2.5.1 (https://bioscience.fi/services/cell-imaging/flowing-software), on the basis of which the necessary calculations of the parameters of the transduced populations were carried out during selection.

Most of the experiments were performed three times with similar outcomes; the figures demonstrate the representative examples of experiments.

## 3. Results

### 3.1. Retroviral Expression Constructs

**Figure 1:**
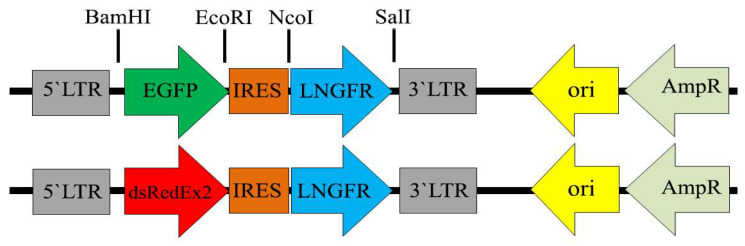
Organization of bicistronic pMigLNR2-EGFP and pMigLNR2-DsREx expression constructs used in this study. The constructs were prepared on the basis of retroviral MigR1 vector via several cloning steps as described in Materials and Methods.

Bicistronic vectors using the internal ribosomal entry site (IRES) provide an excellent opportunity for the creation of transduced mammalian cell populations in which the expression level of the gene of interest directly correlates with the expression level of a selectable marker [22]. To study the magnetic selection of transduced cells, we prepared expression constructs based on MigR1 bicistronic retroviral vector [19], which contain the fluorescent reporter protein ORF in the 1st position of the bicistronic unit and the cell surface selection marker LNGFR in the 2nd position, linked by the IRES of the EMCV virus. Gene expression in these constructs is driven by the viral LTR promoter. Figure 1 shows the schemes of expression constructs used in this study to obtain the stably transduced NIH 3T3 populations.

### 3.2. Magnetic Selection of Transduced of EGFP-Positive Cells with High and Low Doses of MBs

**Figure 2:**
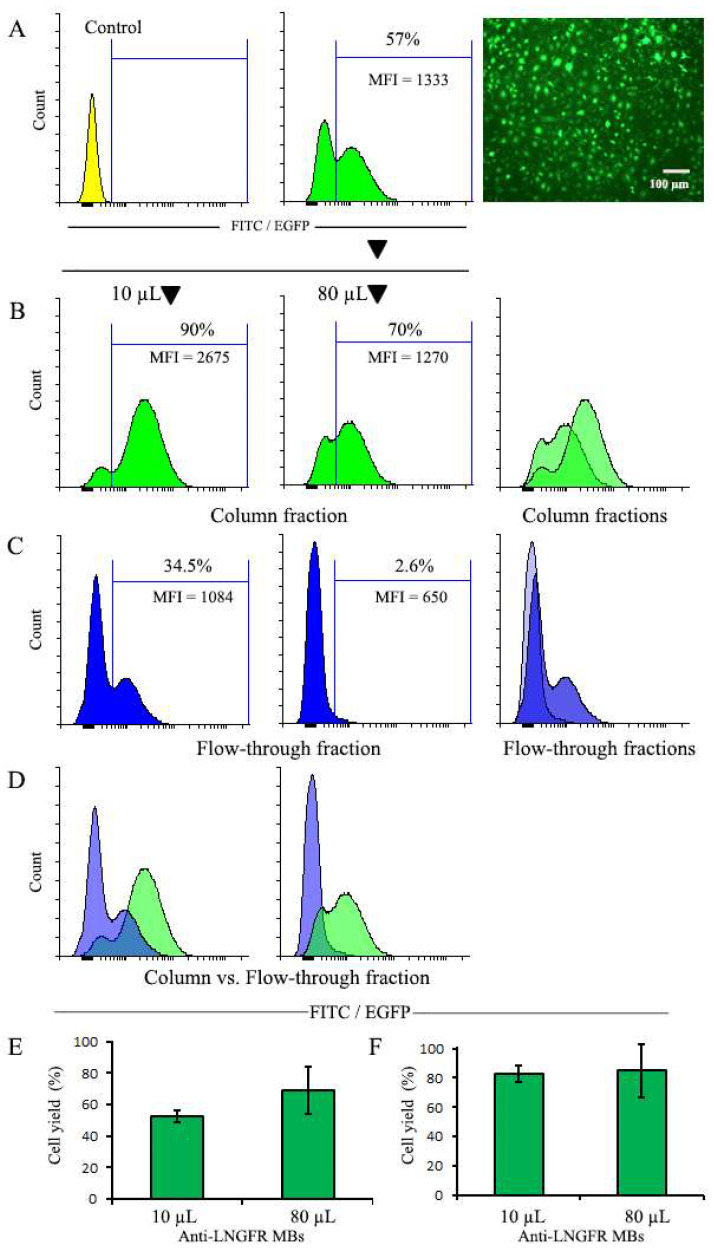
Magnetic selection of transduced cells with 10 µL or 80 µL of MBs. (**A**) Histogram and microscopy of NIH 3T3 cells transduced with pMigLNR2-EGFP construct before magnetic selection; the leftmost diagram depicts the profile of control non-transduced NIH 3T3 cells. (**B**,**C**) Cells of the column-retained and flow-through fractions after selection with 10 µL and 80 µL MBs, respectively. The rightmost diagrams in (**B**,**C**) represent the comparison of profiles of column-retained and flow-through fractions after selection with 10 µL and 80 µL MBs. (**D**) Comparison of profiles of column-retained and flow-through fractions. (**E**) The yield of total cells after selection. (**F**) The yield of EGFP^+^ cells after selection. The number of cells used for selection (2.2 × 10^6^) was taken as 100%.

NIH 3T3 cells were transduced with pMigLNR2-EGFP retroviral supernatant (diluted 1:1 with culture medium), which resulted in a population with 57% EGFP^+^ cells, as evidenced by the cytometry analysis (Figure 2A). The difference in fluorescent levels between EGFP-positive and EGFP-negative populations was modest, with the MFI (median fluorescent intensity) of EGFP-positive cells being only 6.28 times greater than the MFI of EGFP-negative cells. The transduced cell population was used to study the effects of the MB dose on the magnetic selection of cells using magnetic-activated cell sorting (MACS) technology developed by Miltenyi Biotec.

It has already been reported that antibody concentration during magnetic sorting may affect the yield but not necessarily the purity of selected cells [9]. To study this in more detail in our system, we performed a selection of LNGFR^+^ cells with two different doses of LNGFR MBs. Figure 2 shows the result of the selection of EGFP^+^ cells with 10 µL and 80 µL of MBs. Before selection, the EGFP-positive cell population had the MFI value of 1333. As a result of the selection with 10 µL of MBs, the MFI value increased almost 2-fold to MFI = 2675, indicating the preferential selection of cells with high EGFP expression (Figure 2B). After selection with 80 µL of MBs, the MFI of the positive peak changed little, demonstrating the absence of selection towards higher EGFP expression. Importantly, the flow-through fraction for the 80-µL selection variant consisted nearly exclusively of EGFP-negative cells, whereas for the 10-µL selection variant, the flow-through fraction also contained a fraction of EGFP-positive cells characterized by a lower EGFP expression compared to the main EGFP peak (Figure 2C). This apparently resulted in the depletion of the weakly EGFP-expressing cells from the starting EGFP^+^ population, with corresponding elevation of MFI value.

The number of cells in the column-retained fraction after selection with 80 µL MB was a quarter higher than with 10 µL MB, corresponding to the approximately 70% yield (Figure 2E). At the same time, the proportion of selected EGFP^+^ cells relative to the initial EGFP^+^ cell representation differed little (Figure 2F); thus; the yield of EGFP^+^ cells after selection with 10 µL or 80 µL MBs was similar. With 80 µL and 10 µL MBs, the purity of the selected EGFP^+^ population was 70% and 90%, respectively. Thus, magnetic selection with high MB doses yielded an unbiased EGFP-positive population with incompletely depleted negative cells, whereas selection with low MB doses resulted in better removal of negative cells and elevated EGFP expression levels. A brief summary of the main results of the selection experiment depicted in Figure 2 as well as other selection experiments described below is provided in the Supplementary Table S1.

### 3.3. Magnetic Selection of Pre-Selected EGFP-Positive Populations with High and Low Doses of MBs

**Figure 3:**
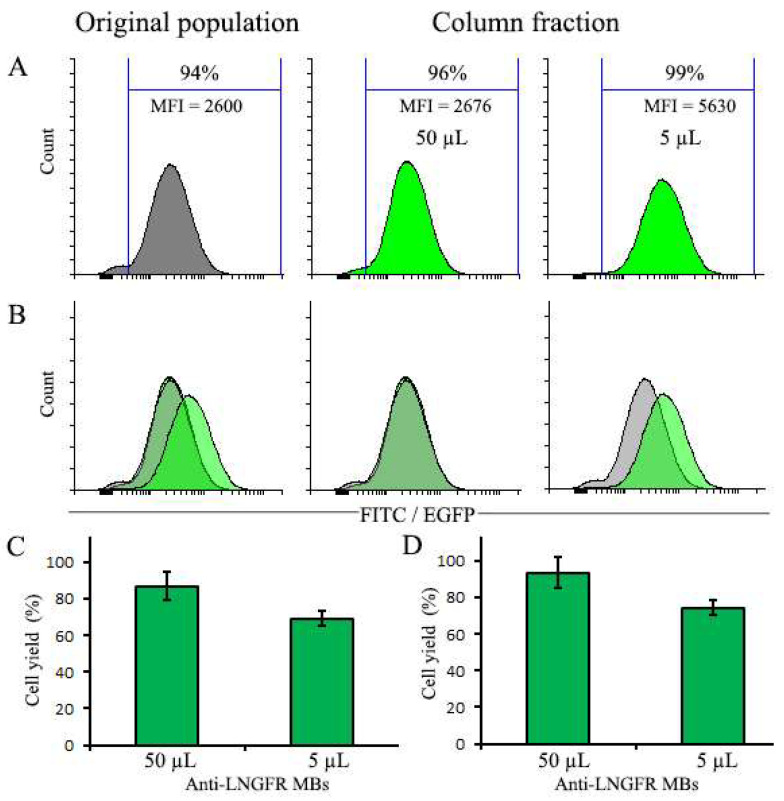
Magnetic selection of pre-selected EGFP-positive NIH 3T3 cells with 5 µL and 50 µL of MBs. (**A**) Cells before (left panel) and after selection with indicated amounts of MBs. (**B**) Combined histograms of cells before and after selection. (**C**) The yield of total cells after selection. (**D**) The yield of EGFP^+^ cells after selection. The number of cells before selection (2.2 × 10^6^) was taken as 100%.

As effects of MB amounts on cell section may be partially obscured by the presence of negative cells, we repeated the selection experiment using a pre-selected cell population. For that, cells selected with 10 µL of MBs (Figure 2) were cultured for 20 days, followed by magnetic selection with 5 µL and 50 µL of MBs. After selection with 50 µL of MBs, the MFI of the cell population remained essentially unchanged, whereas after selection with 5 µL of MBs, the MFI values increased more than 2-fold (Figure 3A). The purity of selected EGFP^+^ cells was in both cases quite high since the starting population was already 94% EGFP-positive. Thus, as in the case of the original transduced cell population, small amounts of MBs promoted the selection of cells with higher EGFP expression, whereas for high amounts of MBs, no such selection occurred.

### 3.4. Selection of EGFP-Positive Cells from Populations Highly Differing in EGFP Expression Levels

#### 3.4.1. Preparation of Transduced Cell Populations Highly Differing in EGFP Expression Levels

**Figure 4:**
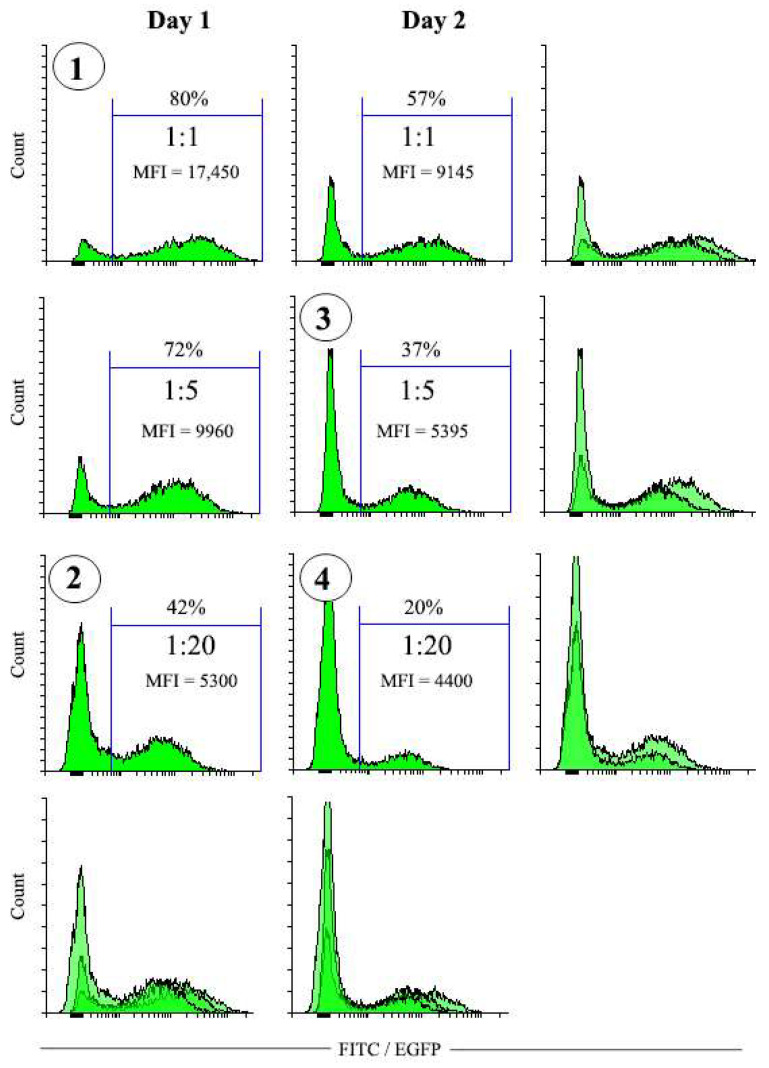
Transduction of NIH 3T3 cells with pMigLNR2-EGFP VCS diluted 1:1, 1:5, and 1:20 with virus collected on the 1st and 2nd days after transfection of HEK 293 cells. Cell populations numbered 1–4 (circled) were used for subsequent selection with 10 µL of MBs (see Figure 5).

The results described above indicate that for cell populations with relatively high percentage of EGFP^+^ cells, the low amounts of MBs favor selection of significantly purer EGFP-positive cell populations compared to the high MB amounts. To test whether the same principle applies to populations with much lower EGFP expression levels and percentages of EGFP-positive cells, we performed transduction of NIH 3T3 cells with various doses of pMigLNR2-EGFP viruses, which resulted in transduced populations with highly differing EGFP expression levels and percentage of EGFP^+^ cells (Figure 4). As expected, the time of virus collection and the dilution factor strongly affected the MFI values and the percentages of EGFP^+^ cells in transduced cell populations. The NIH 3T3 population with the highest 80% EGFP^+^ cell percentage and MFI = 17,450 was obtained following the transduction with virus supernatant diluted 1:1 and collected on the first day after transfection (Figure 4, Table 1). On the other hand, transduction with the virus collected on Day 2 and diluted 1:20 resulted in the cell population with 20% EGFP^+^ cells characterized by the lowest MFI value (4400) (Figure 4, Table 1). Table 1 shows the MFI values of the NIH 3T3-transduced populations. Four of the six cell populations were chosen for experiments with magnetic selection.

#### 3.4.2. Selection of EGFP-Positive Cells from Populations Highly Differing in EGFP Expression Levels

**Figure 5:**
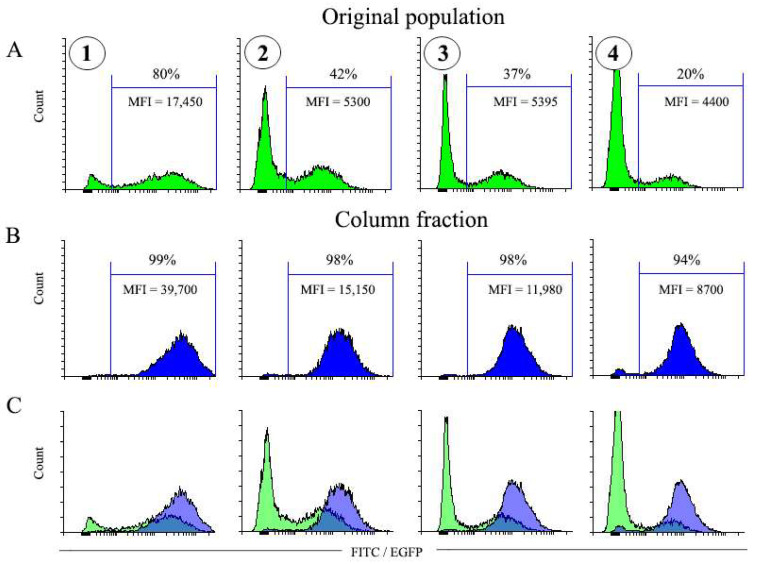
Magnetic selection of four variants of transduced NIH 3T3 cells with 10 µL of MBs. (**A**,**B**) Histograms of cells before and after magnetic selection with 10 µL of MBs, respectively. (**C**) Combined histograms of variants before (green) and after selection (blue). The number of cells before selection (2.2 × 10^6^) was taken as 100%. Transduced cell populations numbered 1–4 (circled, see Figure 4) were taken for selection with 10 µL of MBs.

As described in previous sections, selection with 10 µL of MBs promoted the enrichment of cells with high EGFP expression level (Figure 2). We thus performed magnetic selection of transduced populations with varying EGFP expression levels (Figure 4) using 10 µL MBs. The results of this experiment are depicted in Figure 5. In all cases, the MFI value of the selected populations increased 2–3-fold. The purity of selected EGFP^+^ populations was 98–99%. For a population with the lowest EGFP^+^ cell content of 20%, the selection resulted in a 94% pure population. The obtained results, thus, unequivocally indicate that selection with low amounts of MBs ensures a highly efficient removal of negative cells and a high purity of selected cell populations.

#### 3.4.3. Selection of EGFP-Positive Cells Using Very Small Doses of MBs

**Figure 6:**
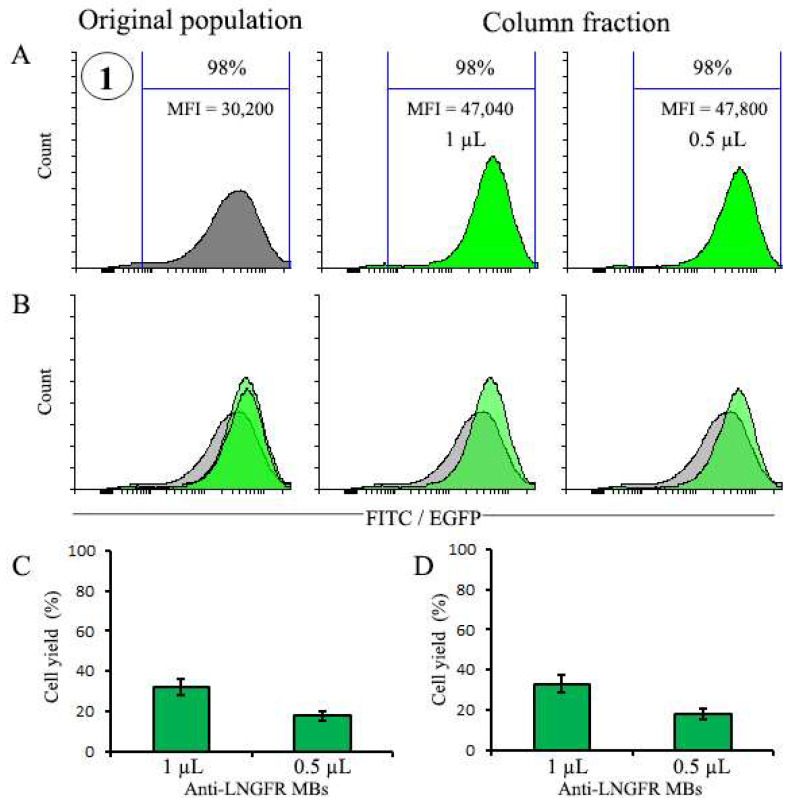
Magnetic selection of pre-selected EGFP-positive cells with very low (0.5 µL and 1 µL) doses of MBs. (**A**) Profiles of populations before and after selection with 0.5 µL and 1 µL of MBs, respectively. (**B**) Combined histograms. (**C**) The yield of total cells after selection. (**D**) The yield of EGFP^+^ cells after selection. The number 1 (circled) is the number of the variant of the cell population pre-selected earlier with 10 µL of MBs (see Figure 5). The number of cells before selection (2.2 × 10^6^) was taken as 100%.

Although selection with small amounts of MBs ensures high purity of the resulting populations, an important question remains as to the effects of further reduction in MBs’ amount on selection process. To address this issue, we used the population with the highest maximum value of MFI = 39,700 from the previous experiment (Figure 5) and cultured it for 25 days, which resulted in the gradual decline of the MFI value to MFI = 30,200. We then performed a magnetic selection of these cells using only 1 µL and 0.5 µL of MBs. The results depicted in Figure 6 demonstrate that these very small amounts of MBs made an additional increase in transgene expression level of already highly expressing cell population feasible, resulting in a nearly 1.6-fold increase in the MFI value. This was, however, achieved at the expense of cell yield since the recovery of cells in column fraction after selection was 32% and 18% for 1 µL and 0.5 µL MBs, respectively.

### 3.5. Two-Stage Magnetic Selection of EGFP-Positive Cells

The data described above indicate that the magnetic selection of cell populations using small doses of MBs results in retention on the column of cells with high transgene expression levels, while the flow-through fraction is enriched in cells with low transgene expression, although also contains a certain proportion of cells with high transgene expression. We reasoned that another round of magnetic selection of the flow-through fraction using greater amounts of MBs may help to remove the high-expressing component, resulting in the separation of the initial heterogeneous cell population into two fractions characterized by low and high expression levels.

**Figure 7:**
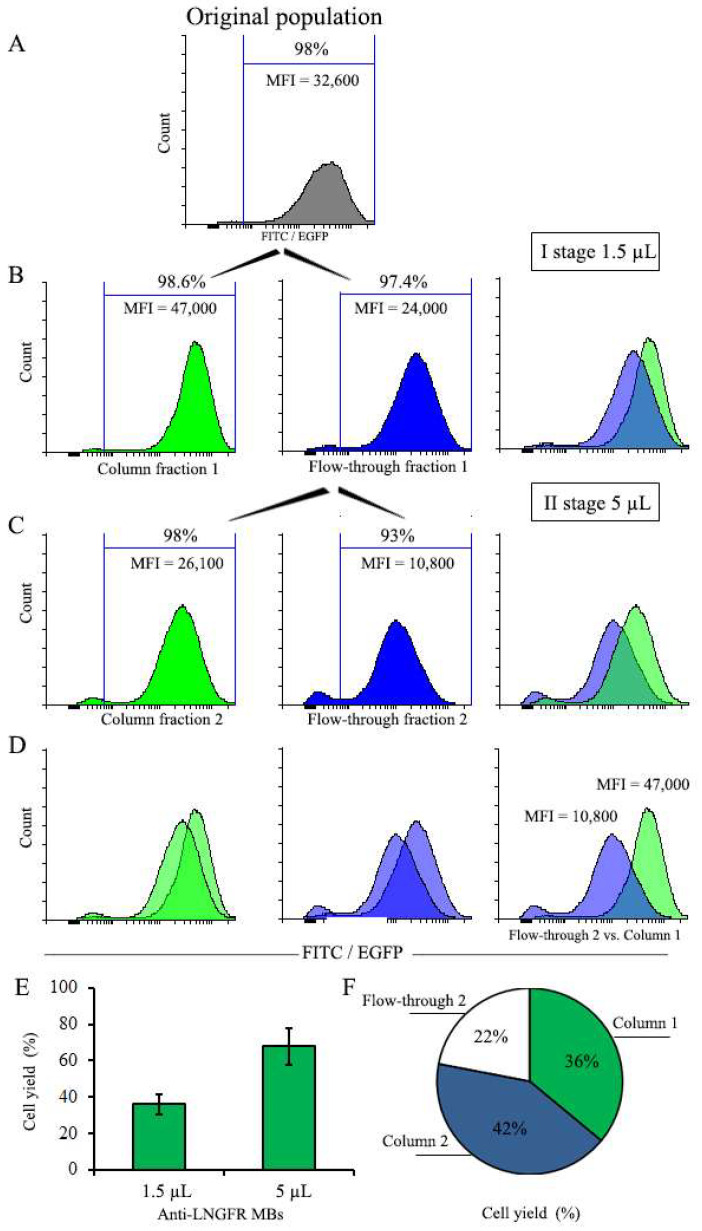
Two-stage magnetic selection of EGFP-positive cells. (**A**) Original cell population before selection. (**B**) Histograms of column-retained and flow-through fractions after 1st stage of magnetic selection with 1.5 µL of MBs. (**C**) Histograms of column-retained and flow-through fractions after 2nd stage of magnetic selection with 5 µL of MBs. (**D**) Combined histograms of the column-retained and flow-through fractions of cells after 2nd stage of magnetic selection. The rightmost diagram represents a comparison of low-expressing (flow-through Fraction 2) and high-expressing (column-retained Fraction 1) cell subsets obtained after two stages of selection. (**E**) Yield of total cells in the column-retained fractions after selection with 1.5 µL and 5 µL of MBs. (**F**) Distribution of total cells in fractions after 2-stage selection. The number of cells before selection (2.5 × 10^6^) was taken as 100%.

To test this, we performed the two-stage magnetic selection of the cell population that was earlier magnetically pre-selected to remove the major part of negative cells. The results of this experiment are presented in Figure 7. At the first selection stage, the starting population was treated with 1.5 µL of MBs followed by column purification. The cell fraction retained on the column had MFI = 47,000, while the MFI of the cells of the flow fraction was nearly 2-fold lower (MFI = 24,000) (Figure 7B). The cells of this flow-through fraction were then treated with 5 µL of MBs and subjected to the second selection round (Figure 7C). As a result of the second selection round, the first flow-through fraction was separated into higher-expressing column fraction and a lower-expressing second flow-through fraction. Importantly, the second flow-through fraction had an MFI value that was about 4.4-fold lower than the first column fraction (10,800 vs. 47,000). These results demonstrate the proof of principle that the two-stage magnetic selection procedure with increasing amounts of MBs is able to separate initial heterogeneous population into subsets with low and high expression levels.

### 3.6. Magnetic Selection of DsRed-Express2-Positive Cells

**Figure 8:**
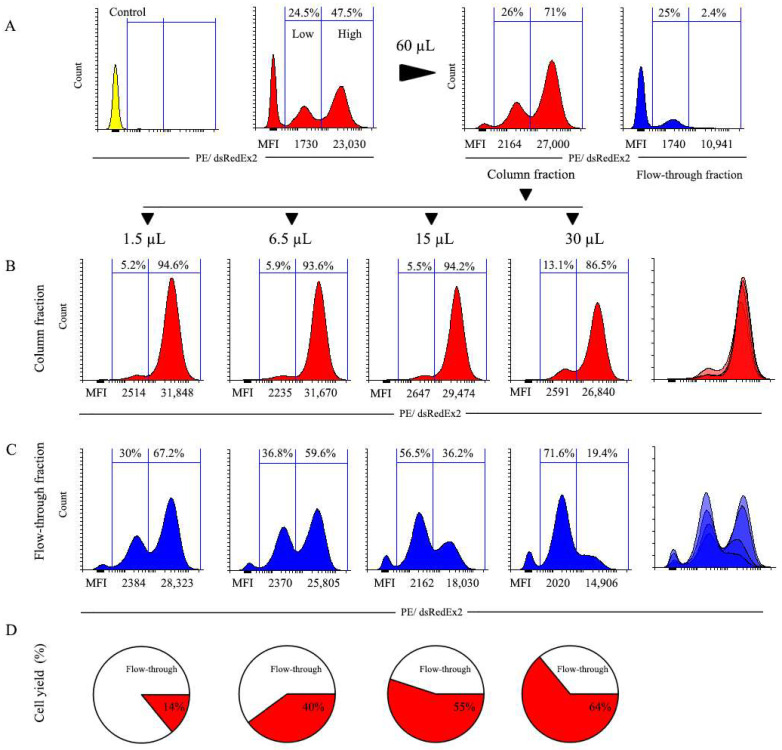
Magnetic selection of DsRed-Express2-positive cells. (**A**) Pre-selection of the original transduced population with 60 µL of MBs. The leftmost diagram depicts the profile of control non-transduced cells (**B**) Column-retained fraction after selection of pre-selected and cultivated positive population with 1.5, 6.5, 15, and 30 µL of MBs. (**C**) Flow-through fraction after selection of pre-selected and cultivated positive population with respective doses of MBs. (**D**) Distribution of total cells in column-retained and flow-through fractions after selection with various doses of MBs. The number of cells used for selection (2.2 × 10^6^) was taken as 100%.

The results of the two-stage selection experiment of the previous section demonstrate that this procedure allows separation of heterogeneous cell population into low- and high-expressing subsets using magnetic sorting. However, despite the evident separation of peaks, the difference in expression levels was modest, with MFIs of two fractions differing by a factor of 4.4. We reasoned that one of the main reasons for this could be a fairly narrow distribution of expression levels in the starting population, which is represented essentially by one relatively tight peak. To test this assumption, we performed another selection experiment with cells transduced with the pMigLNR2-DsREx construct expressing DsRed-Express2 fluorescent protein. According to our previous experience with this construct, it provides for a much broader distribution of expression levels compared to the EGFP-based construct. This is confirmed by Figure 8A, which demonstrates that after transduction, the positive population spans nearly three decades of fluorescent signal intensities and incorporates two cell populations with low and high DsRed-Express2 expression levels (henceforth referred to as low and high cells) representing 24.5% and 47.5% of cells, with MFIs of 1730 and 23,030, respectively.

The starting transduced cell population was first pre-selected with 60 µL of MBs to deplete the population of the majority of negative cells, followed by cultivation of the resulting cell fraction for 14 days. Even with the large quantity of MBs, the preferential selection of high cells was observed, as flow-through fractions contained some low cells but virtually no high ones, which resulted in a relative decline of low peak. Thus, selection with 60 µL of MBs was able to selectively isolate low cells in the flow-through fraction.

The magnetic selection of the pre-selected cultured population with different amounts of MBs was then carried out (Figure 8B). The results of this selection confirmed the previous observations, demonstrating that low MBs amounts promote the selection of high cells and the depletion of the low ones, whereas large doses of MBs provided for a more even selection of high and low cells. At the same time, the increasing MB doses progressively depleted the flow-through fractions of the high cells.

Thus, with higher doses of MBs, it is possible to obtain flow-through fractions containing mostly low cells, as indicated by the results of the selection with 60 µL (Figure 8A) and 30 µL (Figure 8B) of MBs. We conclude that with a proper combination of the low MB doses during the first selection round and high MB doses during the second selection, it would be possible to separate heterogeneous cell populations into fractions containing predominantly high or low cells.

### 3.7. Analysis of Magnetic Selection in EGFP- or DsRed-Express2-Positive Populations Using LNGFR Antibodies

**Figure 9:**
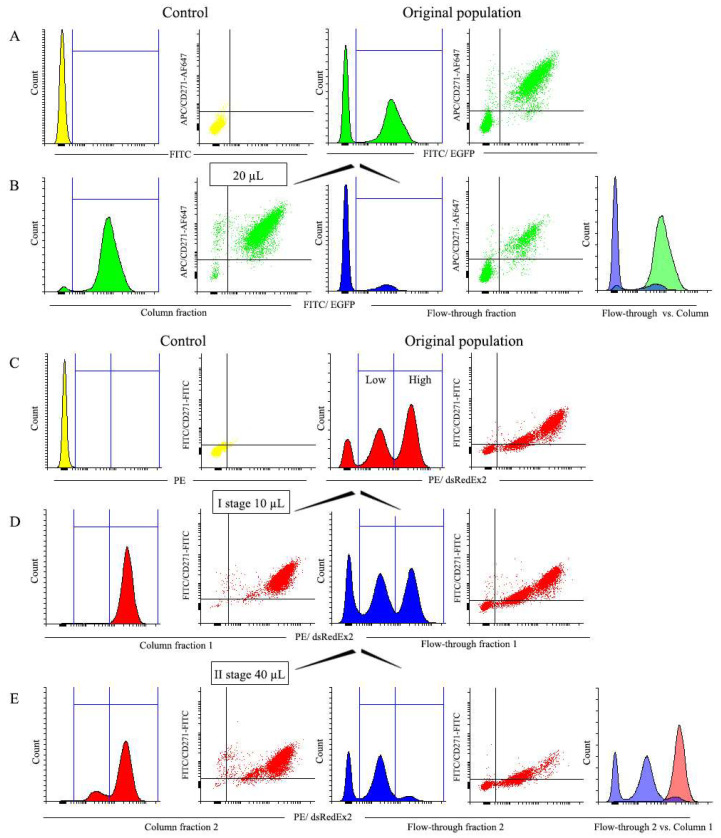
Magnetic selection of EGFP-positive (**A**,**B**) and DsRed-Express2-positive (**C**,**E**) cells analyzed with the use of LNGFR antibodies. (**A**) Non-transduced NIH 3T3 cells (control) and EGFP-transduced cells, unstained (histograms), and stained with CD271-AlexaFluor 647 antibodies (dot plots) before selection. (**B**) EGFP-transduced cells, unstained (histograms), and stained with CD271-AlexaFluor 647 antibodies (dot plots) after selection with 20 µL of MBs. (**C**) Non-transduced cells NIH 3T3 (control) and DsRed-Express2-transduced cells, unstained (histograms), and stained with CD271-FITC antibodies (dot plots) before selection. (**D**) DsRed-Express2-transduced cells, unstained (histograms), and stained with CD271-FITC antibodies (dot plots) after the first stage of selection with 10 µL of MBs. (**E**) DsRed-Express2-transduced cells, unstained (histograms), and stained with CD271-FITC antibodies (dot plots) after the second stage of selection with 40 µL of MBs. The rightmost diagram in row E represents a comparison of low-expressing (flow-through Fraction 2, violet) and high-expressing (column-retained Fraction 1, red) cells subsets obtained after two stages of selection. The number of cells used for selection was 2.5 × 10^6^.

The above described results of magnetic cell selection using LNGFR MBs were based on the analysis of the distribution of EGFP or DsRed-Express2 fluorescent signals in cell populations co-expressing fluorescent protein and LNGFR. Although for bicistronic vectors, the levels of both expressed proteins within a given cell are considered to be highly correlated, the evidence based on fluorescent protein signal distributions in this context should properly be considered indirect. To address this issue, we performed additional magnetic selection experiments to compare the distribution of fluorescent proteins with direct antibody-mediated staining of cell surface LNGFR in resulting populations. For this, antibodies against CD271 (LNGFR) conjugated with contrasting fluorescent dyes were used. Thus, EGFP-positive cells were stained with AlexaFluor 647-conjugated antibodies, while DsRed-Express2-positive cells were stained with FITC-conjugated ones. The results of these experiments are depicted in Figure 9.

In particular, Figure 9A shows that all EGFP-positive cells express LNGFR. After magnetic selection with 20 µL of MBs, cells highly expressing both EGFP and LNGFR are selected in the column fraction (Figure 9B). A similar result was obtained with a two-stage magnetic selection of DsRed-Express2-positive cells. As can be seen in Figure 9C, a fraction of cells not expressing both LNGFR and DsRed-Express2 is present in the initial population. However, after the first selection stage with 10 µL of MBs, the high cell fraction (cells expressing high levels of both DsRed-Express2 and LNGFR) is selected in the Column Fraction 1 (Figure 9D) and is virtually devoid of negative cells. After the second stage of magnetic selection with 40 µL of MBs (Figure 9E), low cells (those with low expression of both DsRed-Express2 and LNGFR) are selected in the flow-through Fraction 2. Therefore, with two-stage magnetic selection of populations characterized by a broad distribution of marker expression, the separation of the initial cell sample into largely non-overlapping cell subsets (as illustrated by the rightmost panel in Figure 9E) is possible. It should be noted, however, that low fraction in this scheme may still contain a substantial number of negative cells, which can be removed by extra selection with a large quantity of MBs.

Thus, the results obtained with the direct LNGFR staining of selected cells clearly demonstrate the high degree of correspondence between separation patterns arising from fluorescent protein analysis and those obtained with LNGFR antibodies, providing a thorough validation of the results described in previous sections.

## 4. Discussion

To assess the effects of various doses of MBs on the outcomes of magnetic selection, we used NIH 3T3 cells transduced with retroviral bicistronic vectors expressing LNGFR as a selection marker and fluorescent reporter proteins EGFP or DsRed-Express2, which allowed a detailed cytometric analysis of the results of retroviral transduction and cell selection. Evidence, including the results of this study, indicates that the efficiency of cell transduction depends on a number of factors, in particular, on the viral dose during the transduction. As a result of transduction, the proportion of marker-positive cells in the population and its expression profile can vary significantly. With low transduction efficiency, the selection of positive cells is usually required, and magnetic selection is considered one of the most effective methods. The attractiveness of magnetic selection technology is associated with a number of advantages of the method [2,3], but this approach also suffers from certain inherent drawbacks [5,6,7], which are partially being counteracted by the development of new approaches and methods for magnetic cell selection [15,16,17,18]. One of the most significant disadvantages of magnetic selection process has long been considered its low discrimination power, which resulted in all-or-none selection outcomes, namely bulk separation of cells populations into positive and negative fractions. The possibility of a more discriminating selection has not been previously discussed in the literature.

The results of this study on the effects of different doses of MBs showed that in all cases, the magnetic selection of cells with the low doses of MBs contributes to the selection of cells with high expression of the selective marker. To the best of our knowledge, this feature of the magnetic selection process has not been previously reported. The depletion of negative cells was also more efficient with low MB doses. At the same time, low MB doses resulted in a reduced yield, primarily due to the selective loss of cells with low marker expression. The MFI values of populations selected with low MB doses may increase by at least a factor of 2 in the case of a fairly narrow range of expression levels, and substantially more in the case of populations with broad expression distribution, as the experiment with DsRed-Express2-positive cells has demonstrated. At the same time, high doses of MBs increase the yield of positive cells and provide a more faithful representation of cells with varying marker expression levels.

These findings have permitted us to separate heterogeneous cell populations into high- and low-expressing subsets. In the case of a rather narrow distribution of expression levels, as with the EGFP vector, the use of a two-stage selection process was necessitated, which resulted in the separation of the initial population into distinct, although partially overlapping, cell subsets, differing in expression levels by a factor of 4.4. It is likely that with the optimization of selection conditions, it will be possible to obtain a better separation of high- and low-expressing subsets. In the case of a wide expression level distribution as with DsRed-Express2, using low and high amounts of MBs was sufficient to obtain a clear separation of high and low cell populations. However, in this case as well, the two-stage selection provided a better separation of subsets with high and low marker expression.

In the study by Kowalewicz-Kulbat et al. in 2016 it was noted that a 2-fold decrease in the concentration of antibodies during the magnetic selection of monocytes did not affect the purity, selectivity, and expression of CD14 in the selected cells [14]. In contrast, in our experiments with transduced cells, we analyzed the effects of up to tenfold and even higher reduction in the MB dose, which clearly favored the selection of cells with high expression. In certain experiments, transduced populations were first pre-selected to remove negative cells, followed by a 2–3 week cultivation, which should be sufficient for the cells to lose their magnetic label acquired after the magnetic selection [23].

Although the analyzed cell samples in the current study represent transduced cell populations, we see no reasons why the described approach for the selection of cells with high or low marker expression cannot be applied to non-transduced immortalized cell lines, provided that the specific surface marker is available for magnetic selection. Moreover, it is likely that this approach can be used for primary cells in case the potential reduction in cell yield is not a critical factor. However, the entailing losses of cells with low marker expression may be disadvantageous for some applications.

It should also be noted that the concentration of antibodies during the magnetic selection may affect not only the number of selected cells, but also the purity of the cell selection. In particular, it was previously noted that the purity of the cell selection during the magnetic selection depends on antibodies supplied by different manufacturers and the selection conditions [5,7,13]. The results of this study demonstrate that small doses of MBs contribute not only to the preferential selection of cells with high marker expression, but also to an increase in the purity of cells selected on the column, even for populations with quite a low percentage of positive cells, as judged by the percentage of negative cells in the selected cell samples. Additional factors that affect the purity of the selected fractions include cell viability and aggregation, the size heterogeneity of the selectable cells, the non-specific mechanical retention of cells on the column matrix, and the selection conditions.

Our results also indicate that the difference in the expression levels of negative and positive cell populations is an important factor strongly affecting the success of the magnetic selection process. With the high expression levels of the selective marker, low amounts of MBs used for magnetic purification favor the selection of purer cell populations. At the same time, the low marker expression levels necessitate the use of high amounts of MBs for selection in order to avoid losses of low-expressing cells. However, our data demonstrate that with high amounts of MBs, the contamination of selected populations with negative cells becomes more pronounced. It is, at present, unclear whether this is the result of a non-specific surface interaction of truly negative cells with MBs, or at least if some of the “negative” cells are not in reality negative but do express very low levels of selection marker, possibly as a result of a variable degree of silencing of integrated viral genomes. We, however, favor the third interpretation, namely that some of the magnetic nanoparticles become non-specifically internalized by cells via the endocytosis pathway, resulting in the labeling of negative cells, which is expected to be proportional to the concentration of MBs during labeling.

Our data also provide a cautionary note, namely that in situations where populations that are to be selected contain subsets of cells with low expression levels, using even high doses of MBs does not guarantee the correct representation of all subsets, and may result in substantial under-representation of low-expressing cells following selection.

## 5. Conclusions

Magnetic cell sorting is currently a widely applied technology with a number of attractive features such as simplicity, independence of expensive equipment and capacity for mass cell selection. However, its applications have been limited so far to the bulk separations of cell populations into largely negative and positive subsets, without further discrimination. In the current study, we developed an approach that provides more selective outcomes of magnetic cell sorting. In particular, cell selection with low doses of MBs results in the depletion of weakly expressing cells while ensuring a better removal of negative cells. Our results further demonstrate that selection with low MB doses results in the enrichment of cells with high expression levels in the column-retained fraction, while high MB doses favor the enrichment of low-expressing cells in the flow-through fraction. This makes the use of magnetic cell sorting possible not only for the specific enrichment of cells with a high expression of selectable marker, but also for the separation of cell populations into subsets with high or low marker expression levels.

Taken together, the results of this study suggest that the proposed approach extends the discriminating power of current magnetic sorting technologies. Given the ability of magnetic sorting to work with large, clinically relevant cell samples, this may open new possibilities in a number of biomedical applications, including the field of regenerative medicine, particularly in those cases where selection of cell populations with high or low expression of specific marker may be advantageous for clinical outcomes. In certain applications, this approach can also significantly reduce the consumption of magnetic beads during selection.

## Figures and Tables

**Table 1:** Parameters of the transduced populations (related to Figure 4).

Time of Virus Collection	Day 1			Day 2		
VCS:medium	1:1	1:5	1:20	1:1	1:5	1:20
MFI(+)/MFI(−)	125.9	91.2	63.1	107.2	61.7	52.5

MFI(+)/MFI(−) value indicates the ratio of MFIs for positive and negative populations for each transduction condition.

## Data Availability

Original data are available upon request.

## Supplementary Materials

The following are available online at https://www.mdpi.com/article/10.3390/cells12091286/s1, Table S1: Main characteristics of fluo-rescent cell populations before and after magnetic selection.
