# Supplementary material for: Selection of Cell Populations with High or Low Surface Marker Expression Using Magnetic Sorting
Source: Cells. 2023 Apr 29;12(9):1286. doi: 10.3390/cells12091286 (PMC10177026; doi:10.3390/cells12091286)
Supplement: Supplementary file 1 [file cells-12-01286-s001.zip › cells-2305640-supplementary.pdf]

Table S1. Main characteristics of fluorescent cell populations before and after magnetic selection.

Relevant figure	Percentage of positive cells	MFI	Percentage of positive cells	MFI	Percentage of positive cells	MFI	Percentage of positive cells	MFI
**Figure 2**								
Before	57	1333						
10 μL MBs	90	2675						
80 μL MBs	70	1270						
**Figure 3**								
Before	94	2600						
50 μL MBs	96	2676						
5 μL MBs	99	5630						
**Figure 5***								
Before	(1)<br>80	(1)<br>17,450	(2)<br>42	(2)<br>5300	(3)<br>37	(3)<br>5395	(4)<br>20	(4)<br>4400
10 μL MBs	99	39,700	98	15,150	98	11,960	94	8700
**Figure 6**								
Before	98	30,200						
1 μL MBs	98	47,070						
0.5 μL MBs	98	47,800						
**Figure 7**	I stage	I stage						
Before	98	32,600						
1.5 μL MBs	98.6	47,000						
	II stage	II stage						
Before	97.4	24,000						
5 μL MBs	98	26,100						
**Figure 8**	Low cells		High cells					
Before	24.5	1730	47.5	23,030				
60 μL MBs	26	2164	71	27,000				

*Results for THE Figure 5 are presented in several columns and correspond to four cell populations transduced with different amounts of viral supernatant.
